# Acoustic and psychoacoustic analysis of the noise produced by the police force firearms

**DOI:** 10.1590/S1808-86942011000200005

**Published:** 2015-10-19

**Authors:** Heraldo Lorena Guida, Thiago Hernandes Diniz, Sérgio Koodi Kinoshita

**Affiliations:** 1 PhD, Assistant Professor; 2 Speech Therapist; 3 PhD, Professor. Faculdade de Filosofia e Ciências, Universidade Estadual Paulista - UNESP Campus de Marília SP

**Keywords:** firearms, police, noise, occupational

## Abstract

Police officers are exposed to impact noise coming from firearms, which may cause irreversible injuries to the hearing system.

**Aim:**

To evaluate the noise exposure in shooting stands during gunfire exercises, to analyze the acoustic impact of the noise produced by the firearms and to associate it with tonal audiometry results.

**Study design:**

Cross-sectional.

**Materials and methods:**

To measure noise intensity we used a digital sound level meter, and the acoustic analysis was carried out by means of the oscillations and cochlear response curves provided by the Praat software. 30 police officers were selected (27 males and 3 females).

**Results:**

The peak level measured was 113.1 dB(C) from a .40 pistol and 116.8 dB(C) for a .38 revolver. The values obtained for oscillation and Praat was 17.9±0.3 Barks, corresponding to the rate of 4,120 and 4,580 Hz. Audiometry indicated greater hearing loss at 4,000Hz in 86.7% of the cases.

**Conclusion:**

With the acoustic analysis it was possible to show cause and effect between the main areas of energy excitation of the cochlea (Praat cochlear response curve) and the frequencies of low hearing acuity.

## INTRODUCTION

Noise, in Portuguese, is a word derived from Latin's *rugitu*, which means large noise. It is acoustically made up of numerous sound waves with anarchically distributed amplitude and phase ratios, causing an unpleasant sensation^1^. Noise can be continuous - when there is no variation in terms of sound pressure nor sound spectrum; floating - when it presents variations in terms of acoustic energy in function of time; or impact noise - with acoustic energy peaks lasting for less than a second, at intervals greater than one second^2^.

Impact noises are usually produced by quick gas expansion, such as that produced by firearms or bomb explosions. These types of sound can reach intensities of 140 dB SPL (sound pressure level) in frequencies around 2 and 3 kHz and, for this reason, they are harmful for human hearing^3^.

When the human ear is exposed to impact noise, at a sound intensity of 120 dB or higher, there is a risk for acoustic trauma. This noise intensity can cause important and abrupt lesions to the cochlea, such as base membrane rupture, tissue and hair cell disarray. Clinically, one of the consequences of impact noise is an immediate and permanent unilateral or bilateral hearing loss, with tinnitus. In some cases, the hearing loss may improve after a few days^4^; however, permanent exposure to intense noise damage the outer hair cells, especially those on the cochlear basal turn, very likely because this is the area of the organ which is more constantly stimulated^5^. The extension and the grade of the hearing damage has a direct relation with the sound pressure intensity, duration in time, frequency and the greater or lower susceptibility of the individual, which can cause changes to the hearing threshold, or noise-induced-hearing-loss (NIHL) which, just like the acoustic trauma, is irreversible.

NIHL is a sensorineural hearing loss, which initially involves the frequency range between 3 and 6 kHz, where the 8 kHz has to be better than the worst threshold (3, 4 or 6 kHz)^6^, ^7^. This loss affects mainly the cochlear hair cells, situated at about 5 to 10 mm off the vestibular window, exactly on the region receiving the 4 to 6kHz stimuli. The causes for the greater vulnerability of this region have not yet been fully explained, but they may be associated with the resonance characteristics of the outer ear (OE) and of the middle ear (ME), the mechanical and anatomical characteristics of the cochlea and also its blood supply^8^.

The Occupational Hygiene Standard^9^ from Fundacentro shows a formula used to calculate the quantity of impact pulses to which the worker is exposed in each day of work, according with the magnitude of the pulse. Regulatory standard 15 (NR-15) also verses that the worker should not be exposed to sound pressure levels above 130 dB(C)^2^.

One of the industries which expose its workers to high noise levels is the military, especially when practicing with firearms. The firearm explosion noises are among the number one causes of noise-induced hearing loss in the United States^10^.

Auditory profile studies with military personnel in Brazil have shown high rates of hearing loss in this population, and such fact is associated with the excessive exposure to impact noise without individual protection equipment^11^, ^12^, ^13^.

In a study about the auditory profile of military personnel from the Brazilian Army, carried out by means of an interview, otoscopy and audiometric exam, we observed audiometric changes which were supposedly induced by noise in 38.1%, with a predominance of unilateral hearing loss. We also noticed that 64.5 % of the military people examined did not use proper hearing protection^14^.

Other authors measured the level of noise at the workplace of the police force of Paraná, and found sound intensities higher than 100 dB SPL^15^. Within this line of research, we studied auditory thresholds of 22 police officers from Bauru - SP. Of these, 17 had altered audiograms, with notches in the high frequencies^16^.

In the Singapore Armed Forces, they studied the effects of basic military training on hearing. The researchers studied the audiometries of 85 military personnel before and after training, and found a prevalence of 9.4% of hearing loss, which was kept the same after one year. The study allowed us to conclude that the hearing protection program of the Singapore armed forces is efficient and preserves the auditory health of its officers^17^.

A recent study involved the audiological profile of five police officers from the Montes Claros Police Force - MG, Brazil. In checking sound pressure levels, the mean level found at the shooting stand was 97.4 dB and of the five police officers studied, two had audiometric curves with a notch, one unilateral and the other was bilateral, and one showed mild sensorineural hearing loss in the high frequencies with a drop-shaped audiometric curve^18^.

The international literature suggests the need for a hearing preserving program for police officers and military personnel because of the high sound intensities to which this population is exposed to in their work environment^19^, ^20^.

There is a great need to implement a hearing preservation program (HPP) to prevent noise-induced hearing loss, when the noise in the work environment exceeds 85 dB (A) during an 8-hour working shift, for continuous or intermittent noise, or 120 dB (C), for impact noise. Within this program, the first factors to be considered are: environment recognition and noise exposure quantification^21^.

All the previously described papers quantified the intensity levels or sound pressure (SIL ou SPL) from the firearm noise, to which these people are exposed during practice and the relationship of this exposure to the audiometric changes found among the officers examined; however, without a study of the physical characteristics of this type of noise and its influences on the types of hearing loss studied.

The literature has only a handful of studies associated with the acoustic analysis of non-verbal sounds. There are studies which analyze the temporal and spectral representation of the sound stimulus^22^, or which group sounds according to the physical events which generate them, advocating the assumption that the acoustic properties depend on the material (solid, liquid or gas) and the interaction of the materials themselves (e.g., impact, drip or explosion)^23^. However, to the extent of our knowledge, the specific acoustic analysis of the noise produced by firearms, as well as a later comparison with the audiologic findings, is rare in the literature.

Recently in the USA, a study was carried out to estimate the auditory risk of shooting civil firearms. Different types of weapons were analyzed: rifle, shotgun, .38 revolver and 9mm pistol. The maximum peaks obtained in the study were 141 a 164 dB (SPL). Considering the revolver and the pistol, in the analysis of the acoustic spectrum, the peaks of greatest energy were concentrated in the low and medium frequencies, with decreases in the high frequencies^24^.

The influence of the noise spectrum in the prevalence of NIHL among workers was carried out in plants with noise levels above 85 dB. The authors did not find associations between the frequency bands with intense noise levels and the hearing damage injury^25^.

As far as the sounds of musical instruments are concerned, the acoustic measures of duration, intensity and frequency of resonances are the ones most often used in the acoustic analysis of these stimuli^22^. Three periods are considered:

• *Transitional attack:* corresponds to the passage of silence to sound and in most musical instruments it lasts between some milliseconds and some hundredths of a second; it is very important for timbre recognition, and if cut, the instrument becomes non-classifiable in most of the cases;
• *Period of stability:* time between the previous and the following; usually corresponding to the period of a few tenths of a second to some seconds; it is extremely important for certain sound characteristics such as volume and intensity;
• *Extinction transitory (drop):* period in which the sound is extinguished; it is very important for recognizing timbre, and if cut, the instrument becomes non-classifiable in most of the cases.

During the gunshot noise study period, the stability is more important than the other parameters because of duration, intensity and volume in which the soldier will be exposed to this type of sound.

Considered fundamental to associate the exposure of military personnel to firearm noise, the goal of the present study was to assess noise exposure at the shooting stand of the police force, to analyze the acoustic spectrum of the noise produced by the firearms, transforming these values into the Bark scale, in order to compare these data with the audiometric results.

## MATERIALS AND METHODS

We analyzed noise levels from the firearms during gun shooting practice in the Police Academy of the State of São Paulo, analysis from the noise of the firearms used during the shooting practice, and measures of tonal audiometry from a sample of the population of military personnel of this squad. We chose the two weapons which are most frequently used by these officers: .38 revolver and .40 pistol; usually called 38 revolver and .40 pistol, respectively.

This study was submitted to the Ethics Committee in Research, having been duly approved (protocol CEP # 2762/2007), and the procedures realized are in compliance with Resolution 196/1996, from the National Health Council.

### Noise Level Measurement

Noise measurement was done by means of a digital decibelimeter from MINIPA, model MSL - 1350, programmed in the fast response circuit (fast), “C” weighted, according to NR-15, in its Attachment #II, and measure range between 65 and 130 dB. The guns used were the .38 revolver and the .40 pistol, both from Taurus Co.

During training in the shooting booth, the number of participating police officers varied between 12 and 15, and each individual shot the gun 50 times. Thus, multiplying the number of shots by the number of individuals, we calculate from 600 to 750 impact pulses per period of training.

The police officers are duly equipped with Individual Protection Equipment (IPE.), according to NR 620, from Ordinance 3214/78^2^, being relevant for our study to stress the use of proper ear protectors, approved by the Ministry of Labor and Employment.

### Acoustic and Psychoacoustic Analysis

In order to analyze the acoustic spectrum we used a portable DAT recorder coupled to a stereo microphone. The recordings were digitalized in a sampling rate of 44,000 Hz with the use of CSL from *Kay Elemetrics* and analyzed through the *Praat*^26^application. We recorded 10 samples of gunshot sounds with two shooting sequences (5 from .38 revolvers and 5 from .40 pistol). In order to visualize the acoustic spectrum analysis in an integral way, during data collection the recorder volume was set in order to reduce the recording intensity in 50% of the current power.

**Figure 1:**
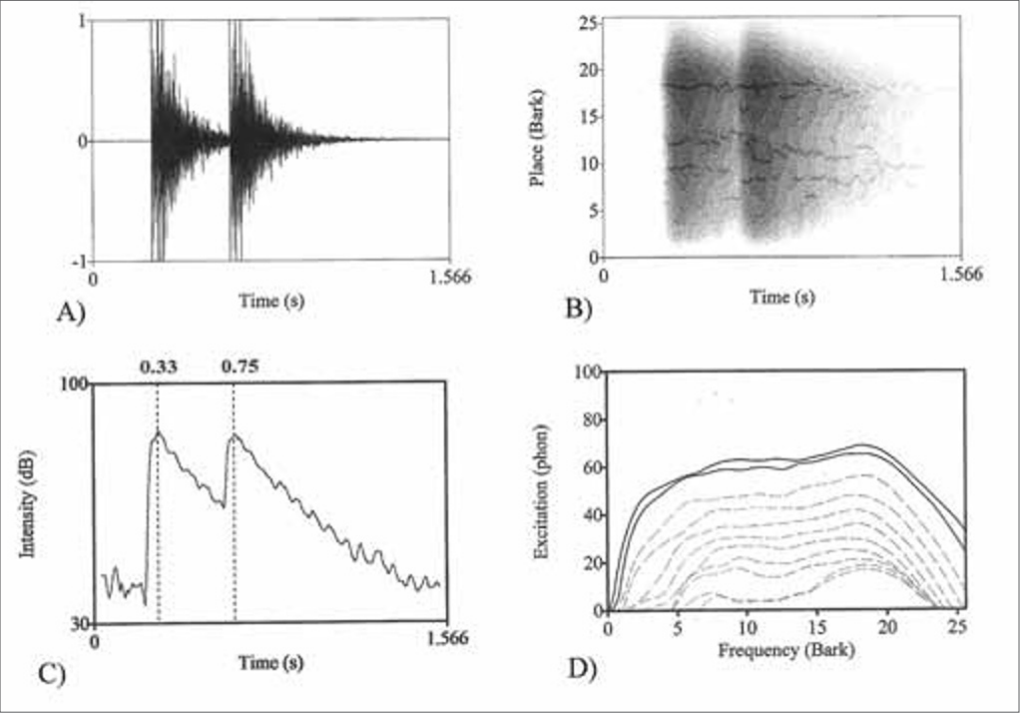
*Praat pictures* from the .38 revolver second sample (Table 1); A - temporal graph (oscillograph); B - Bark temporal position cochleograph; C - sound intensity level temporal graph (dB) with maximum values in 0.33s and 0.75s; D - spectral excitation graph (*fon scale*) where more external continuous lines correspond to a greater degree of excitation for time instants: 0.36s and 0.67s for the two shooting sequences of the sample.

Since the goal of research work was to relate the military exposure with the level of noise submitted to during the training period we adopted the following procedures:

We first analyzed the wave form graphs from the shooting sounds in relation to their temporal representation (Figure 1a). The graph provides information on sound duration and intensity variation throughout time. Based on this *Praat* graph, we plotted the temporal graph of the sound intensity level (dB) (Figure 1b) and we established the peak instants for each shot sequence by means of a matrix of data available at the *Praat* application.

After the temporal acoustic analysis of the shots from the fire weapons, we studied the positional cochleograms (a graph which represents the inner ear base membrane excitation pattern in function of time) from each shooting sequence in order to establish in which regions of the cochlear base membrane there was a higher degree of deflection generated by the noise intensity. Since each region corresponds to a resonance frequency range, such as the Critical Band, it is supposed that the regions are subjected to a higher likelihood of damaging the hair cells because of the high gunshot intensity (Figure 1c). The Critical Band represents a given frequency in the scale between 0 and 24 and a unit in Bark. The position cochleograms enables one to graphically visualize the regions of higher excitation by the gunshot sound; however, it makes it difficult to establish the exact position in which there is the highest degree of excitation. In order to improve the precision of this measure, we raised excitation spectral curves in an iterative way within the time period between 0.1 and 1.5 seconds with 0.01 second intervals. This process of semi-automatic iteration enabled us to establish the time periods in which there is the highest level of excitation (*fon* scale) in the two gunshot sequences (Figure 1d).

With that, it was possible to compare the peak instants obtained from the temporal graphs (wave form and sound intensity level) and the time instants in which there is greater base membrane excitation from the cochleograms (position cochleograms and spectral excitation curve). If the instants are statistically similar, we can assume that instant as the one with the greatest base membrane excitation. These values were established through the data matrix of the graphs available at the *Praat* application. We employed the *t-student* statistical test in order to assess the relationship between the two groups of independent samples with a 5% significance level (a = 0.05) for the null hypothesis (Ho). Knowing these instances of greater excitation, we can raise the Barks band in which this excitation happens and, with that, we establish its frequency range.

**Figure 2:**
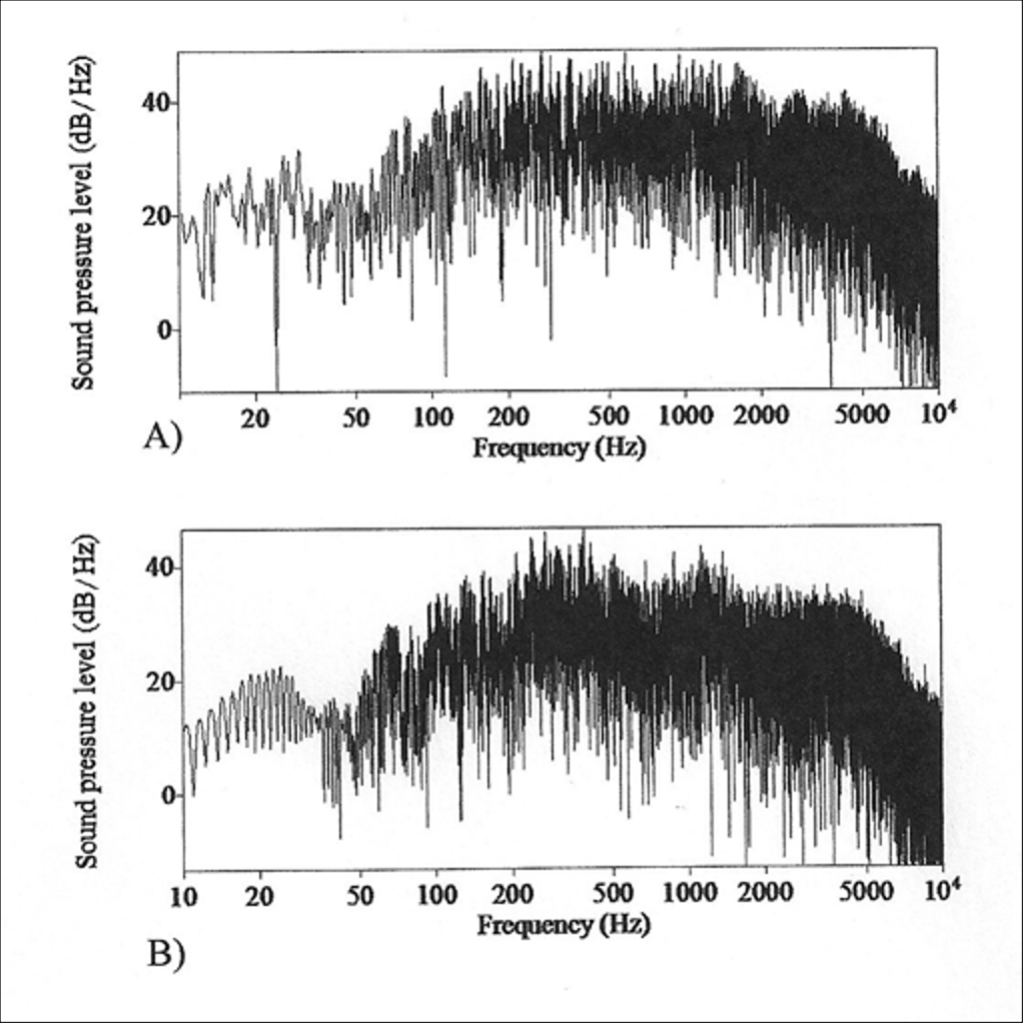
*Praat pictures* - sound pressure level density spectral graph of density distribution (dB): A - sample from the .38 revolver; B - sample from the .40 pistol.

Thus, it was possible to also identify the spectral shape curve of the gunshot sound from the two types of firearms in order to analyze and discuss the transitional period of the attack, stability and extinction transitional. This curve is relevant in order to determine the mean energy density in function of frequency (Figure 2).

### Auditory Threshold Assessment

The auditory threshold measures were carried out in an acoustic booth, with a GSI 61 *Grason - Stadler*^27^ audiometer. For this study we selected the medical records from 30 police officers from both genders (3 females and 27 males), with a mean age of 41.9 ± 3.21 years (34 to 47 years) and mean time in the police force of 19.4 ± 4.02 years (9 to 25 years).

We have also assessed the asymmetry of the audiological values between the tonal audiometry thresholds of the right and left ears using the *McNemar* test starting from the threshold ratios. We used the non-parametric test for two ratios, which assesses the level of disagreement of a variable between two paired samples. The unilateral alteration (left or right ear) is what we call disagreeing pairs. The significance level used was 1% (a = 0.01) for the null hypothesis (H_0_).

## RESULTS

The background noise on the measurement days was 75.3 dB (C), in average, and the maximum peaks measured were 113.1 dB(C) for the .40 pistol and 116.8 dB(C) for the .38 caliber revolver. The noise level mean values were 114.8 dB(C) and 111.9 dB(C), respectively, for the revolver and the pistol.

The time instants in which we had the highest noise intensity in the temporal scale and the greatest excitation in the spectral analysis by means of the cochleograms for the two shooting sequences are depicted on Table 1. The *t-student* test employed between the two groups of analyses resulted in *p*=0.29 (*p*>a=0.05) for the 1st shot and *p*=0.17 (*p*>a=0.05) for the 2nd shot, confirming the null hypothesis (H_o_), in other words, there were no significant differences in the results comparison between the times of the two samples (temporal form *versus* cochleograph).

**Table 1:** Time values in seconds, corresponding to the sound intensity level curve peaks (dB) and the curves of greater cochleograph excitation for the two sequences of shots from 10 samples of weapons.

Weapon	Temporal Form Analysis		Cochleograph Analysis	
	Time (s)		Time (s)	
	1st shot	2nd shot	1st shot	2nd shot
.38 Revolver	0.48	1.03	0.45	0.89
	0.33	0.75	0.36	0.67
	0.19	0.60	0.24	0.54
	1.39	1.85	1.24	1.60
	1.50	1.94	1.27	1.60
.40 Pistol	1.52	2.56	1.29	2.11
	1.04	1.92	0.91	1.62
	1.08	1.86	0.93	1.60
	1.76	2.42	1.50	1.99
	1.24	1.98	1.10	1.65

Thus, the statistical test guaranteed the use of the excitation peak time values from the cochleograph (Table 1) as a reference to establish the Bark scale in which there was the highest degree of base membrane excitation (Table 2). The values were established on the Bark scale for the 10 samples with two shooting sequences. For the .38 revolver, the Bark scale was in 18.1 and 18.2 in average for the 1^st^ and 2^nd^ shot, respectively. For the .40 pistol, the Bark scale was 17.8 and 17.6 in average for the 1^st^ and 2^nd^ shots, respectively. Therefore, we can consider that in average, for the two types of weapons, the value was around 17.9±0.3 Barks, corresponding, by linear interpolation, to a cochlear stimulation area between 4120 and 4580 Hz.

**Table 2:** Frequency range obtained from the base membrane excitation pattern in the Bark scale for the 10 samples of sounds from the weapons (in two sequences of shots) analyzed by the positional cochleograph and the excitation graph.

Weapon	Shot 1- Shot 2	Frequency range	
	(Bark scale)	(Hz)	
.38 Revolver	17 - 18	3700	5300
	18 - 18	4400	5300
	18 - 18	4400	5300
	17 - 18	3700	5300
	18 - 18	4400	5300
.40 Pistol	18 - 18	4400	5300
	17 - 18	3700	5300
	18 - 18	4400	5300
	17 - 17	3700	4400
	18 - 18	4400	5300

The spectral form (Figure 2) has a descending shape for both types of weapons, with an energy drop after the 5,000 Hz frequency. We consider that, despite the similarities between both types of acoustic spectra, the attack transitional period (which happens near 200 to 250 Hz) and the stability period (range between 300 and 5,000 Hz), with a mildly increased tilt on the .40 pistol spectral analysis curve when compared to the acoustic spectrum of the .38 revolver shot.

The data obtained in the tonal audiometry test of this sample of police officers, depicted on Table 3, showed a reduction in their hearing acuity above 30 dB(HL) in the frequency range between 3,000 and 6,000 Hz, with a greater acuity loss in the frequency of 4,000 Hz (86.7%), depicting a notch-like hearing loss. The lower hearing acuity losses happened to the frequencies below 3,000 Hz (30.0%) and 8,000 Hz (36.6%). The bilateral hearing losses had higher values for the frequencies of 3,000 Hz and 4,000 Hz, not going beyond 36.7% of the total sample; the unilateral range, in these frequencies did not go beyond 16.7% (RE) and 33.3% (LE), and the normal bilateral in 13.7%. The McNemar statistical test employed resulted in accepting the null hypothesis (Ho) with a lower *p* value = 0.22 (*p*>a=0.01), statistically confirming the symmetry of the hearing alteration in this sample.

**Table 3:** Distribution of the audiometric alteration measures in a sample of 30 officers from the police force population, unilateral RE, unilateral LE and bilateral, considering the range of time spent in the service.

f (kHz)	Audiometric alteration	Time in Service (years)				Total	Total	Total
		0 -10	11 - 15	16 - 20	21- 25	N	%	%
	RE	0	0	1	1	2	6.7	
< 3,0	LE	0	0	1	3	4	13.3	30,0
	Bilateral	1	0	0	2	3	10.0	
	RE	0	1	1	1	3	10.0	
3,0	LE	0	0	3	5	8	26.7	56,7
	Bilateral	0	1	3	2	6	20.0	
	RE	0	1	1	3	5	16.7	
4,0	LE	2	0	5	3	10	33.3	86,7
	Bilateral	0	2	5	4	11	36.7	
	RE	0	1	1	1	3	10.0	
6,0	LE	1	0	2	3	6	20.0	66,7
	Bilateral	0	2	5	4	11	36.7	
	RE	0	2	1	1	4	13.3	
8,0	LE	0	0	3	1	4	13.3	36,6
	Bilateral	0	0	2	1	3	10.0	

RE: right ear; LE: left ear; N: number of cases with audiometric alterations.

## DISCUSSION

During practice with firearms, we identified at least 600 sound impacts caused by firing the weapons. Since the Norma Técnica Fundacentro^3^ establishes the maximum level of noise for these impacts to be 109 dB (C), it is clear the unhealthy situation lived by these professional during their practice.

Other authors also found high levels of sound intensity in the occupational environment of police officers^7^, and studies of audiological assessment of police officers found high levels of hearing loss in this population^5^.

The data hereby presented is in agreement with those in the national and international literature, concerning the identification of an unhealthy occupational environment and point to the need to create a hearing protection program for these law enforcement professionals^3^, ^10^, ^15^, ^16^, ^19^, ^20^.

The temporal (Figure 1a) and spectral (Figure 2) analyses findings concerning the sounds from the firearms showed a high energy density in a broad frequency range and short time duration. We noticed that the transitional attack period happens near 200 to 250 Hz, the stability period is within the 300 and 5000 Hz range, and the extinction transitional period starts at 5,000 Hz. The duration of the pulses is less than one second and the high intensity of noises near 120 dB(C) enabled us to preliminarily assume these police officers had their hearing acuity altered.

The acoustic and psychoacoustic analyses of the gun shooting sounds pointed to some relevant aspects on the cause-and-effect analyses concerning the hearing loss detected in this sample of police officers. It established, *a priori*, the regions in which there was a higher degree of cochlear basal membrane excitation with this type of noise, enabling the establishment of corresponding frequency ranges. In the Praat cochleograph analysis it was possible to notice an intense concentration of energy within 17.9±0.3 Barks, in other words, in the sensitivity region of the high cochlear frequencies: 4120 and 4580 Hz.

Audiometric findings corroborated what was presented in the Praat cochleograph, since the frequency with the worst auditory thresholds was the 4,000 Hz (Table 3). Therefore, these policemen had the sensorineural notchlike hearing loss^28^, the classical noise-induced hearing loss type^6^, ^7^.

The data presented in the acoustic and audiometric analyses confirm that the permanent exposure to noise damage the outer hair cells, especially those on the cochlear basal turn, very likely because this is the part of the organ which is most commonly stimulated^8^.

Although the statistical results on the hearing loss asymmetry between the ears (right and left) lead us to accept the null hypothesis (Ho), we have noticed that the proportion of hearing loss is slightly higher on the left ear for all frequencies, except 8,000 Hz - in which the proportion is the same (Table 3). This fact is associated with the head position during the shooting and it is relevant during the creation of a hearing protection program with the use of proper hearing protection devices for each ear^29^.

The measures and analyses carried out enabled the recognition of the environment and the quantification of the noise exposure^21^, and, in a complementary fashion, it was also possible to establish the reference audiogram for the police officers by means of audiological evaluations. Considering the need for continuity in audiometric management in this population, we will continue this work, with notices about the harmful effects of noise and the periodic follow up of the hearing situation of each police officer.

Concerning the acoustic analysis, the present study found results which were similar to those reported from the United States: the highest energy peaks were concentrated in the low and medium frequencies, with a drop in the high frequencies^24^. Thus the psychoacoustic analysis importance: it enables a more accurate correlation of the noise influence on the cochlea.

Another study reports on the relationship between the noise acoustic spectrum and NIHL; however, the authors did not do a psychoacoustic analysis to prove their results^25^. Thus, we do not corroborate this data, which describes the intensity as the main cochlear lesion factor, despite the acoustic spectrum.

## CONCLUSION

The present study identified sound pressure levels which were higher than the ones recommended by the Fundacentro NHO 01 standard, which establishes that the maximum limit for the population studied should not go above 109 dB(C).

Based on the acoustic and psychoacoustic analyses of the gun shots, it was possible to show that there is a coincidence between the main areas of energy concentration (*Praat's cochleograph*) on the high frequencies, and the audiometric findings show the cause and effect relation between the firearm shots and the hearing loss.

Thus, we conclude for the need of developing a hearing protection program for the police officers in order to prevent the onset, as well as the worsening, of hearing loss in this population.
